# Did the effect of placebo increase in rcts of panic disorder across the years?

**DOI:** 10.1192/j.eurpsy.2021.1627

**Published:** 2021-08-13

**Authors:** F. Di Segni, T. Zoppi, F. Forcina, G. Anibaldi, P. Bargagna, C.L. Telesforo, F. Montebovi, G. Callovini, G. Giuseppin, D. Janiri, M. Molinaro, G. Sani, G.D. Kotzalidis

**Affiliations:** 1 Nesmos Department (neurosciences, Mental Healt And Sensory Functions), Sant’andrea Hospital, “Sapienza” University of Rome, Rome, Italy; 2 Psychiatry, ASL Roma 1, Rome, Italy; 3 Psychiatry, ASL Latina, Latina, Italy; 4 Psychiatry, ASL Rieti, Rieti, Italy; 5 Ageing, Neurosciences, Head-neck And Orthopaedics Sciences, Fondazione Policlinico Universitario Agostino Gemelli IRCCS, Università Cattolica del Sacro Cuore, Rome, Italy; 6 Psychiatry, Sapienza University, Rome, Italy; 7 Department Of Neuroscience, Section Of Psychiatry, Università Cattolica del Sacro Cuore, Rome, Italy

**Keywords:** drugs, panic attack, panic disorder, Placebo

## Abstract

**Introduction:**

The curious effect of an increase of the placebo effect across year of publication has been shown for depression, schizophrenia, obsessive-compulsive disorder, as well as for some medical conditions like hypertension and pain.

**Objectives:**

We aimed to observe how randomised clinical trials with a placebo control behave at this respect in panic disorder trials.

**Methods:**

We searched the PubMed database using the strategy: (panic disorder OR panic attack disorder) AND placebo, which on 3 November 2020 produced 779 records. Inclusion criteria were the above stated, excluded were all studies focusing on the same patients as others and those not providing intelligible data. In our selection we used the PRISMA statement and reached agreement with Delphi rounds.

**Results:**

We identified through other sources further 3 studies. The finally eligible studies were 82, excluded were 700 studies, mainly consisting of reviews (176), challenge studies (173), not dealing with panic disorder (67), studies with unsuitable designs to detect placebo effect (53), studies using same populations as others (36), those with misfocused outcomes (57), those lumping diagnoses and not allowing to separate data for panic disorder (22), and those not using placebo at all (21). Mean response to placebo in included panic disorder studies was 36.01±19.812, ranging from 0 to 76.19%; the correlation with year of publication was positive and significant (Pearson’s r= 0.246; p=0.026).

**Conclusions:**

The effect of placebo in randomised control trials has increased across the years, but this field of research appears to be idle in recent years.

**Disclosure:**

No significant relationships.

